# ELDER COHOUSING: THE EMBODIMENT OF AGING IN COMMUNITY

**DOI:** 10.1093/geroni/igz038.1520

**Published:** 2019-11-08

**Authors:** Anne P Glass

**Affiliations:** University of North Carolina Wilmington, Wilmington, North Carolina, United States

## Abstract

A new alternative living arrangement has emerged in the U.S., in which older adults proactively choose how, where, and with whom they want to live, in a close-knit community where neighbors look out for each other. Adopting the cohousing model originally established in northern Europe, these elder intentional communities are distinctive, as they are run by the residents themselves, and there is a focus on neighbors helping each other. Drawing from over a decade of research incorporating data from six communities, the challenges and benefits of establishing such a community will be addressed and the requirements necessary for mutual support to thrive will be identified. Finally, the model of aging better together intentionally, developed from the senior cohousing experience, will be shared, as well as the implications for how the model can be used for planning and policy in other settings.

